# A Comprehensive Survey of Deep Learning Approaches in Image Processing

**DOI:** 10.3390/s25020531

**Published:** 2025-01-17

**Authors:** Maria Trigka, Elias Dritsas

**Affiliations:** Industrial Systems Institute (ISI), Athena Research and Innovation Center, 26504 Patras, Greece; trigka@isi.gr

**Keywords:** image processing, deep learning, techniques, models, metrics

## Abstract

The integration of deep learning (DL) into image processing has driven transformative advancements, enabling capabilities far beyond the reach of traditional methodologies. This survey offers an in-depth exploration of the DL approaches that have redefined image processing, tracing their evolution from early innovations to the latest state-of-the-art developments. It also analyzes the progression of architectural designs and learning paradigms that have significantly enhanced the ability to process and interpret complex visual data. Key advancements, such as techniques improving model efficiency, generalization, and robustness, are examined, showcasing DL’s ability to address increasingly sophisticated image-processing tasks across diverse domains. Metrics used for rigorous model evaluation are also discussed, underscoring the importance of performance assessment in varied application contexts. The impact of DL in image processing is highlighted through its ability to tackle complex challenges and generate actionable insights. Finally, this survey identifies potential future directions, including the integration of emerging technologies like quantum computing and neuromorphic architectures for enhanced efficiency and federated learning for privacy-preserving training. Additionally, it highlights the potential of combining DL with emerging technologies such as edge computing and explainable artificial intelligence (AI) to address scalability and interpretability challenges. These advancements are positioned to further extend the capabilities and applications of DL, driving innovation in image processing.

## 1. Introduction

The field of image processing has been revolutionized by the advent of DL, a subset of AI inspired by the structure and processes of the human brain to analyze and interpret complex data patterns. Traditionally, image processing relied heavily on manual feature extraction and classical machine learning (ML) techniques, which required significant domain expertise and often struggled with the variability and complexity inherent in visual data. These methods, while effective in specific, well-defined tasks, lacked the flexibility and scalability needed to handle the diverse and high-dimensional nature of real-world images [1,2,3].

DL, characterized by its ability to learn hierarchical representations directly from raw data, has addressed many of the limitations of traditional approaches. The introduction of multi-layered neural networks (NNs) enabled models to automatically discover intricate patterns and features that were previously unachievable with manual techniques. This shift from handcrafted feature engineering to automated feature learning marked a pivotal moment in image processing, allowing for significant advancements in both accuracy and generalizability across a broad range of applications [4,5,6].

One of the most significant breakthroughs in DL was the ability to process large-scale image datasets, which provided the foundation for developing robust and generalizable models. These models not only excelled in traditional image processing tasks, such as classification and segmentation, but also opened new avenues for innovation in areas that were previously considered too challenging or computationally prohibitive. The availability of large datasets and the increase in computational power, particularly through the use of Graphics Processing Units (GPUs), further accelerated this progress, making DL the dominant paradigm in image processing [7,8,9].

The architectural advancements in DL models have also played a crucial role in this evolution. The design of more complex and deeper networks, capable of capturing a wide range of visual features across different scales, has enabled the processing of images with unprecedented accuracy. These models have evolved to handle various aspects of image processing, from low-level tasks like denoising and super-resolution to high-level tasks such as object detection and semantic segmentation. Each new generation of models has built upon the successes of its predecessors, incorporating novel mechanisms to enhance learning efficiency, reduce computational costs, and improve model interpretability [10,11,12].

Moreover, the versatility of DL has facilitated its application across numerous domains, demonstrating its ability to solve complex and domain-specific challenges. The adaptability of DL models to different types of visual data—from natural images to medical scans—has led to breakthroughs in diverse fields, significantly impacting research and industry practices alike. This has established DL not just as a tool for solving image processing problems but as a fundamental technology driving innovation across a wide spectrum of scientific and technological endeavours [13,14,15].

Despite these advancements, the application of DL in image processing is not without challenges. The reliance on large, labelled datasets raises concerns about the scalability of these models to tasks where annotated data are scarce or difficult to obtain. Additionally, the high computational demands of training deep networks, particularly as models grow in complexity, pose significant barriers to entry for many researchers and practitioners. The interpretability of DL models also remains a critical issue, especially in high-stakes applications wherein understanding the model’s decision-making process is as important as its accuracy [16,17,18].

The rapid advancements and widespread adoption of DL in image processing have led to an explosion of research, resulting in a vast and fragmented body of knowledge. As new models and techniques continuously emerge, it becomes increasingly challenging for researchers and practitioners to stay abreast of the latest developments and to understand how these advancements interrelate. This survey is motivated by the need to consolidate and synthesize this growing body of work, providing a structured and comprehensive overview that can serve as both a reference for current researchers and a guide for future exploration. Furthermore, while many reviews focus on specific aspects of DL in image processing—such as particular models or applications—there is a need for a broader survey that not only covers the evolution of models but also delves into the underlying techniques, evaluation metrics, and emerging trends. By addressing these elements, this survey aims to bridge the gap between theory and practice, offering insights that are relevant across a range of applications and research contexts. Specifically, this survey makes several key contributions to the field of DL in image processing:

- We provide an in-depth examination of the evolution of DL models in image processing, from foundational architectures to the latest advancements, highlighting the key developments that have shaped the field.
- The survey synthesizes various DL techniques that have been instrumental in advancing image processing, including those that enhance model efficiency, generalization, and robustness.
- We discuss the critical metrics used to evaluate DL models in image processing, offering a nuanced understanding of how these metrics are applied across different tasks.
- This survey identifies the persistent challenges in the application of DL to image processing and also explores potential future directions, including the integration of emerging technologies that could further advance the field.

**Figure 1:**
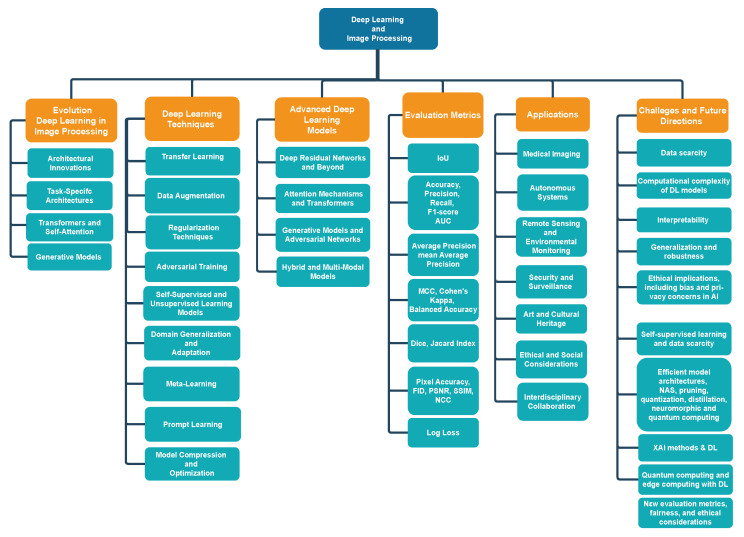
An overview of surveyed key topics in image processing with DL.

The remaining paper is illustrated in Figure 1 and is structured as follows. Section 2 provides the evolution of DL in image processing. Moreover, Section 3 describes DL techniques in image processing. Section 4 notes advanced DL models. Next, in Section 5, the evaluation metrics for image processing models are provided. Section 6 presents applications of DL in image processing. Moreover, Section 7 illustrates challenges and future directions. Finally, Section 8 concludes the present survey.

## 2. Evolution of Deep Learning in Image Processing

The evolution of DL in image processing represents a transformative journey from rudimentary NN models to modern architectures capable of handling complex visual data with unparalleled accuracy. This evolution is marked by several pivotal developments that have redefined the capabilities of image processing systems, pushing the boundaries of what was once thought possible in the field.

### 2.1. Architectural Innovations

The architectural evolution in DL for image processing has been pivotal in addressing the complex challenges posed by high-dimensional visual data. Convolutional NNs (CNNs) laid the groundwork by effectively capturing spatial hierarchies through convolutional layers [19]. However, the introduction of deeper networks, such as residual networks (ResNets), marked a significant leap forward. ResNets leverage residual connections to bypass one or more layers, mitigating the vanishing gradient problem and enabling the training of exceptionally deep networks. This advancement allows these models to learn richer and more intricate features, leading to substantial improvements in tasks such as image classification and object detection [20,21]. Furthermore, the densely connected convolutional network (DenseNet), with its densely connected layers, further enhances this capability by promoting feature reuse across layers, reducing the number of parameters required, and improving both computational efficiency and model accuracy [22,23,24].

Multi-branch architectures, exemplified by inception networks, represent another significant development, enabling models to capture information at multiple scales within the same architecture. This design allows the network to process various feature scales simultaneously, enhancing its ability to generalize across different image-processing tasks. Such architectures are particularly effective in handling the diverse and complex nature of visual data, making them ideal for advanced tasks like semantic segmentation and image synthesis. The integration of these architectural innovations has not only pushed the boundaries of what DL models can achieve but also set new standards for performance in the field of image processing [25,26,27,28].

The field of object detection has seen remarkable progress with the introduction of models like YOLO (You Only Look Once), which revolutionized real-time detection by using a single NN to predict bounding boxes and class probabilities simultaneously. Unlike traditional methods that rely on region proposals, YOLO’s unified approach significantly reduces computational complexity while maintaining accuracy, making it a preferred choice for applications requiring speed and efficiency. Its ongoing development, from YOLOv1 to YOLOv8, demonstrates its adaptability and continued relevance in DL research [29,30].

The next generation of convolutional networks (ConvNext) is a modernized CNN that integrates design principles from vision transformers while retaining the simplicity and efficiency of traditional CNNs. It revisits standard convolutional architectures and improves them with innovations like depth-wise convolutions, layer normalization, and expanded kernel sizes, achieving competitive performance in image classification, object detection, and segmentation. ConvNext bridges the gap between CNNs and attention-based architectures, combining the strengths of both approaches [31,32,33].

### 2.2. Specialized Architectures for Task-Specific Challenges

As DL models evolved, the need for specialized architectures tailored to specific image-processing tasks became apparent. Fully convolutional networks (FCNs) and U-Net architectures were developed to address the challenges of pixel-level predictions required in semantic segmentation. FCNs replace fully connected layers with convolutional layers, maintaining spatial hierarchies and enabling dense prediction tasks [34]. U-Net, with its encoder–decoder structure and skip connections, is particularly effective in capturing both contextual information and fine-grained details [35]. These features make U-Net highly suitable for medical imaging and other applications wherein precise boundary delineation is critical [36,37].

In object detection, mask region-based convolutional NNs (R-CNN) have set a new benchmark by extending the capabilities of region-based CNNs to include pixel-level segmentation [38]. This architecture integrates detection and segmentation tasks within a unified framework, enabling comprehensive scene understanding. The ability to generate high-quality segmentation masks for detected objects has proven invaluable in applications requiring detailed scene analysis, such as autonomous driving and video surveillance [39]. The development of these specialized architectures underscores the importance of designing task-specific solutions to meet the growing demands of advanced image processing challenges [40,41,42].

### 2.3. Expanding Capabilities with Transformers and Self-Attention

The introduction of self-attention mechanisms, particularly through vision transformers (ViTs), has expanded the capabilities of DL models in image processing. Unlike traditional CNNs, which focus on local features through fixed convolutional filters, transformers model global dependencies within an image. This capability allows ViTs to capture long-range relationships that are crucial for understanding complex scenes, making them particularly effective in tasks that require holistic image analysis, such as scene segmentation and object recognition. The scalability of transformers, which can be achieved with minimal architectural changes, makes them well-suited for handling large and diverse datasets [43,44,45,46,47].

Self-attention mechanisms have also paved the way for more flexible and powerful models. By dynamically focusing on different parts of an image based on task relevance, these models can prioritize critical features while ignoring irrelevant data. This selective attention mechanism enhances the model’s ability to generalize across varied image-processing tasks. As transformers continue to evolve, their integration into hybrid architectures that combine the strengths of both CNNs and transformers is likely to yield even greater performance gains, further pushing the boundaries of what DL models can achieve in the field of image processing [48,49,50,51,52,53].

### 2.4. Integration of Generative Models

Generative models, particularly generative adversarial networks (GANs), have introduced new dimensions to DL in image processing. GANs have revolutionized the field by enabling the generation of high-quality, realistic images through a competitive training process involving two networks: the generator and the discriminator. This framework allows GANs to learn complex data distributions without explicit probabilistic modeling, making them highly effective for tasks such as image synthesis, style transfer, and super-resolution [54,55,56,57]. Advanced variants of GANs, like conditional GANs (CGANs) [58] and Wasserstein GANs (WGANs) [59], have further refined the generative process, addressing challenges such as mode collapse and ensuring more stable training.

Beyond their generative capabilities, GANs have significantly impacted other areas of image processing, such as data augmentation and domain adaptation. In scenarios where labeled data are scarce, GANs can generate synthetic data that closely resemble real-world samples, improving model robustness and generalization. Additionally, GANs are used in domain adaptation to align feature distributions between different domains, facilitating the transfer of models across diverse imaging contexts. The versatility and effectiveness of GANs in enhancing image-processing tasks underscore their importance as a core component of modern DL frameworks [60,61,62,63,64].

Finally, diffusion models have emerged as state-of-the-art generative models, excelling in image synthesis, denoising, and restoration by employing a probabilistic framework to reconstruct data from noise. These models work by gradually adding random noise to data during the forward process and then learning to reverse this process to generate high-quality outputs. This unique approach allows diffusion models to produce highly realistic and diverse data, often surpassing traditional GANs in terms of stability and output quality [65,66].

As illustrated in Table 1, the evolution of DL architectures in image processing has been marked by pivotal innovations. This table summarizes key references that outline the advancements in architectural innovations, scale-aware networks, and task-specific designs, highlighting the impact of DL on various image-processing challenges. The references serve as a foundation for understanding the significant strides made in the field, from foundational models to specialized architectures tailored for complex tasks.

## 3. Deep Learning Techniques in Image Processing

The rapid advancements in DL have been driven not only by the evolution of NN architectures but also by the development of sophisticated techniques that optimize these models for specific image-processing tasks. These techniques are critical to enhancing the capabilities of DL models in terms of accuracy, efficiency, and generalization. This section explores several key techniques that have profoundly impacted the field.

### 3.1. Transfer Learning

Transfer learning has become a cornerstone in DL, particularly within image processing, where it addresses the challenges of training models on limited datasets by leveraging pre-trained models on large, diverse datasets. The principle behind transfer learning is to utilize the feature representations learned from a source task and apply them to a target task, effectively reducing the need for extensive labelled data and computational resources.

According to [67], deep transfer learning approaches can be categorized into two main types: adversarial-based and network-based methods. Adversarial-based techniques leverage adversarial learning strategies to enhance performance. On the other hand, network-based approaches include fine-tuning, freezing CNN layers, and progressive learning, enabling the adaptation of pre-trained models to new tasks by varying the level of layer adjustments and optimization.

The concept of transfer learning is particularly valuable in domains like medical imaging [68], where acquiring large labelled datasets is costly or impractical. By fine-tuning pre-trained models on smaller, task-specific datasets, practitioners can achieve significant performance improvements, often surpassing models trained from scratch [69,70]. The authors in [71] emphasized the application of transformer-based pre-trained models for image processing tasks such as super-resolution and denoising. Leveraging transformers’ ability to capture global dependencies, and adapt to task-specific needs, trained on a large-scale synthetic dataset, the model demonstrated exceptional generalization across diverse tasks. Moreover, the combination of transfer learning with pre-trained CNNs [72] especially in medical image classification is analyzed in [73], focusing on approaches like feature extraction and fine-tuning. It provides actionable insights for leveraging transfer learning to address data scarcity, including recommendations on optimal model selection and configuration. Previous works highlight the transformative potential of pre-trained models in various image-processing tasks.

However, while transfer learning offers many benefits, it is not without challenges. One such challenge is negative transfer, where the knowledge from the pre-trained model may not always be beneficial and could even hinder performance on the target task. This occurs when the source and target tasks are dissimilar, leading to ineffective feature reuse [74,75]. Several categories and approaches to mitigate negative transfer in transfer learning are detailed in [76]. These categories include data transferability enhancement, with methods like domain-level, instance-level, feature-level, and class-level strategies. Model transferability enhancement focuses on techniques like transferable batch normalization, adversarial training, multiple models, parameter selection, and parameter regularization. Training process enhancement involves hyper-parameter tuning and gradient correction. Target prediction enhancement includes soft labeling, selective labeling, weighted clustering, and entropy regularization. Moreover, concept-wise fine-tuning was presented in [77], addressing the problem by maximizing mutual information for rare features and applying causal adjustment to correct spurious correlations, enhancing transfer robustness and effectiveness. Concept-wise fine-tuning falls under model transferability enhancement, emphasizing parameter selection and regularization. These approaches collectively enhance transfer robustness and minimize the impact of irrelevant or harmful knowledge.

A recent study in [78] suggested an approach based on hierarchical transfer progressive learning (HTPL), demonstrating its effectiveness in addressing negative transfer by progressively fine-tuning knowledge from source to target tasks. It begins with transferring general low-level features, which are less domain-specific, before gradually incorporating high-level features tailored to sonar image characteristics such as low resolution and speckle noise. This staged adaptation minimizes the risk of transferring irrelevant knowledge, ensuring effective domain alignment. Apart from its contribution to negative transfer mitigation, it also enhances fine-grained feature extraction, addresses the scarcity of labeled data with self-supervised pre-training, and resolves class imbalance using key point sensitive loss. These strategies demonstrate the solution’s robustness in sonar image classification challenges. A summary of topics discussed regarding transfer learning techniques is presented in Table 2.

### 3.2. Data Augmentation

Data augmentation is crucial in DL, especially in image processing, where the diversity and volume of training data significantly influence model performance. By systematically applying a series of transformations to the original dataset, data augmentation increases the effective size of the dataset and enhances the model’s ability to generalize by exposing it to a broader range of variations and distortions. This technique is particularly important in preventing overfitting, especially when acquiring large datasets is impractical or expensive [79,80,81,82,83].

Advanced data augmentation strategies have evolved beyond basic geometric transformations like rotation and scaling [84]. Techniques such as Cutout [85], Mixup [86], and CutMix [87] introduce more complex variations by blending different image samples or masking out regions of images, encouraging the model to focus on global context rather than specific localized features. The advent of automated data augmentation methods, such as AutoAugment and RandAugment, represents a significant leap forward. These methods use reinforcement learning (RL) and optimization algorithms to automatically discover the most effective augmentation strategies tailored to the specific dataset and task at hand. This reduces the manual effort involved and consistently results in superior model performance, particularly in complex image-processing tasks [88,89,90]. Table 3 provides a comprehensive summary of the techniques addressed on data augmentation.

### 3.3. Regularization Techniques

Regularization techniques are essential for DL models, particularly in image processing, where high dimensionality and complexity of visual data often lead to overfitting which occurs when a model learns not just the underlying patterns in the training data but also the noise and irrelevant details, resulting in poor generalization to unseen data. Various regularization techniques to address overfitting and enhance generalization in DL models are systematically reviewed in [91]. Also, it compares traditional and modern methods, evaluating their computational costs and impact on model performance, with experimental insights to guide practical applications. Key regularization strategies include dropout, weight decay, and batch normalization. Apart from the $L_{2}$ regularization (i.e., weight decay), it explores variations in dropout, like DropAll, Curriculum Dropout, and DropMaps to address co-adaptation and improve model robustness.

Dropout [92,93] is a widely used technique that randomly deactivates a subset of neurons during each training iteration, forcing the network to learn redundant representations of features. This reduces the risk of co-adaptation between neurons and enhances the network’s robustness [94]. Weight decay [95] adds a penalty term to the loss function based on the magnitude of the network’s weights, discouraging the model from assigning excessive importance to any particular weight and thus preventing overfitting. Ref. [96] explores disharmony issues between weight decay and weight normalization methods, offering insights into balancing these regularization strategies. Batch normalization [97], while primarily designed to stabilize and accelerate training, also functions as a regularization technique by reducing internal covariate shift, allowing for higher learning rates and improving overall model performance. The combination of these techniques ensures that models trained on complex image datasets are more likely to generalize well to new data [98]. An overview of the covered techniques related to regularization is provided in Table 4.

### 3.4. Adversarial Training

Adversarial training has emerged as a critical technique in DL, particularly for improving the robustness and security of models in image processing. This approach involves deliberately introducing adversarial examples—inputs subtly perturbed to deceive the model—into the training process. The goal is to fortify the model against potential vulnerabilities by exposing it to these adversarial inputs, thereby enhancing its resilience to attacks that could exploit weaknesses in its predictive capabilities [99,100,101,102,103].

However, generating effective adversarial examples that are both imperceptible to humans and capable of misleading the model remains a challenge. Techniques such as the Fast Gradient Sign Method (FGSM) [104] and Projected Gradient Descent (PGD) [105] have been developed to create these adversarial examples efficiently. These methods perturb input data minimally yet significantly affect the model’s output, challenging the model to learn more robust and invariant representations. Beyond improving robustness, adversarial training has broader implications, such as enhancing the model’s understanding of data distributions and improving generalization.

As research in adversarial training continues, it aims to strike an optimal balance between robustness and accuracy, making DL models more secure and reliable for real-world applications. Free adversarial training reduces the computational cost of adversarial training by reusing gradient computations through minibatch replays, updating both model weights and input perturbations simultaneously. It achieves adversarial robustness comparable to PGD training but with significantly fewer gradient calculations, making it computationally efficient [106]. Universal projected gradient descent (UPGD) is an enhanced adversarial attack method that generates universal perturbations effectively across multiple models and datasets by refining perturbations iteratively. It balances robustness and accuracy, achieving higher fooling rates and cross-model generalization compared to traditional techniques [107]. Finally, model-based adversarial training (MAT) extends traditional adversarial training by leveraging models of natural variation (such as changes in lighting, weather, or resolution) to craft adversarial examples that simulate realistic shifts in data distribution. MAT is directly tied to adversarial training, bridging the gap between adversarial robustness and natural shift generalization [108]. A summarization of the methods in the topic of adversarial training is made in Table 5.

### 3.5. Self-Supervised and Unsupervised Learning

Self-supervised and unsupervised learning has emerged as a transformative approach in DL, particularly for image processing tasks where labeled data are scarce or expensive to obtain [109]. Unlike traditional supervised learning, which relies on large, manually annotated datasets, self-supervised learning leverages vast amounts of unlabeled data by generating proxy tasks that can be solved without human intervention [110]. These proxy tasks enable the model to learn useful representations from the data, capturing images’ underlying structure and semantics. The learned representations can then be fine-tuned for specific/downstream tasks such as classification, segmentation, or detection, often yielding performance that rivals fully supervised methods [111,112,113,114,115,116].

Recent innovations in self-supervised learning, such as contrastive learning, have shown remarkable success in this area [117]. Contrastive learning techniques like the simple framework for contrastive learning of visual (SimCLR) [118] and Momentum Contrast (MoCo) [119] enable models to learn robust representations by maximizing agreement between different augmentations of the same instance. This approach significantly reduces the dependency on labeled datasets, making DL more accessible and scalable across various domains. The integration of self-supervised learning with other advanced techniques, such as attention mechanisms, promises to further enhance the capabilities and applicability of DL in image processing [120,121,122].

Unsupervised learning, which focuses on uncovering the intrinsic structure of data without any explicit labels, has also seen significant advancements: in clustering using the Uniform Manifold Approximation and Projection (UMAP) dimensionality reduction technique [123], dimensionality reduction [124]. In addition to the previous advances, 3D convolutional autoencoders [125] have been instrumental in learning compact, latent representations of images that preserve essential features while reducing noise and redundancy. The recent success of contrastive learning, a method that maximizes the similarity between different augmentations of the same image, has further pushed the boundaries of what can be achieved with minimal supervision. These approaches are particularly valuable in fields like medical imaging, where labeled data are limited, and are likely to become increasingly important as the field progresses [126,127,128]. An extended list of techniques and their purpose of use is concisely captured in Table 6.

### 3.6. Domain Generalization and Adaptation

In image processing, domain variability poses a significant challenge when models trained on one dataset fail to perform adequately on another due to differences in data distribution, a problem known as domain shifts. These shifts can arise from variations in lighting, resolution, imaging devices, or environmental conditions. For example, satellite images captured under varying weather conditions or medical images from scanners with differing configurations often exhibit discrepancies that hinder model generalization [129].

Domain generalization [130] aims to train models that perform robustly on unseen domains without direct access to their data during training. This is achieved by encouraging models to learn domain-invariant features—representations that capture the essence of the data while disregarding domain-specific variations. Techniques such as deep domain confusion (DDC) [131] and domain-invariant component analysis (DICA) [132] are widely used to align feature distributions across multiple source domains. For instance, in medical imaging, a domain-generalized model trained on datasets from diverse hospitals can classify anomalies in scans from a new hospital, even if the imaging protocols differ. Episodic training frameworks, where models simulate potential domain shifts during training, further enhance robustness by preparing the model for unseen variations [133].

Domain adaptation, by contrast, assumes access to target domain data, albeit often unlabeled, during training. This enables explicit alignment of the source and target domain distributions to improve performance on the target domain. A popular approach is adversarial learning, implemented in models like domain-adversarial NNs (DANN) [134]. Here, a domain classifier guides the feature extractor in producing domain-agnostic representations, ensuring the model generalizes well to both domains. Another powerful tool is CycleGAN, which employs style transfer to transform target domain images into the appearance of the source domain [135]. For example, CycleGAN has been used to adapt object detection models for autonomous vehicles, enabling them to perform effectively in rural settings despite being trained in urban environments. The style transfer process aligns lighting, textures, and other visual properties between the domains, ensuring consistent detection accuracy [136]. In conclusion, Table 7 briefly presents the key techniques discussed in this section.

### 3.7. Meta-Learning

Meta-learning, or “learning to learn”, has emerged as a transformative approach in image processing, addressing the challenge of limited labeled data by enabling models to adapt quickly to new tasks. Unlike traditional DL, which requires extensive datasets, meta-learning trains models on diverse tasks to optimize their ability to generalize with minimal data [137].

There are three state-of-the-art types of meta-learning methods for image segmentation: metric-based, model-based, and optimization-based [138,139]. Metric-based approaches, such as prototypical/prototype networks, classify new data points by comparing them to learned class prototypes. Other methods in this category include siamese NNs and matching networks, which rely on feature extractors, similarity metrics, and automatic algorithm selection.

Model-based approaches aim to adapt to new tasks by changing the model’s learnable parameters. For example, memory-augmented NNs (MANNs) combine NNs with external memory modules to enhance learning efficiency. Despite their advantages, MANNs are complex, and meta-networks are computationally intensive with high memory requirements. Alternatively, the simple neural attentive meta-learner (SNAIL) offers a relatively straightforward structure but requires optimization for automatic parameter tuning and reduced computational demands [140].

Optimization-based approaches treat meta-learning as an optimization problem, aiming to extract meta-knowledge that improves optimization performance. These methods generate classifiers capable of performing well on a query set with only a few gradient updates. Model-agnostic meta-learning (MAML) is a widely used method in this category, fine-tuning model parameters for rapid adaptation. Other notable methods include META-LSTM and META-SGD, which leverage long short-term memory (LSTM) and stochastic gradient descent (SGD), respectively. Finally, Reptile, similar to MAML, adapts to new tasks by learning optimal initial parameters but is better suited for problems requiring numerous update steps. With lower variance, it achieves faster convergence but has primarily been validated for few-shot classification, with limited evidence for tasks like regression or RL [138]. To sum up, in Table 8, we capture the meta-learning categories and methods presented previously.

### 3.8. Prompt Learning

Prompt learning is an emerging and impactful paradigm in image processing, enabling pre-trained models to adapt to specific tasks with minimal fine-tuning by embedding task-specific “prompts” into the input data [141]. Inspired by its success in natural language processing, prompt learning has demonstrated its utility in vision-language models like Contrastive Language-Image Pretraining (CLIP) [142,143]. CLIP interprets textual prompts such as “a photo of a deforested area” or “a picture of an urban landscape” to classify images, making it highly versatile for applications like environmental monitoring and disaster assessment [144].

In interactive segmentation, click prompt learning allows for the real-time refinement of outputs. For example, interactive tools utilize user-provided prompts, such as clicks or bounding boxes, to guide segmentation tasks, a technique particularly valuable in medical imaging [145,146]. Also, a Promptable and Robust Interactive Segmentation Model (PRISM), with visual prompts aiming for precise segmentation of 3D medical images, is suggested in [147]. A systematic review and taxonomy of deep interactive segmentation of medical images is thoroughly described in [148], identifying key methods, models, and trends within the field while thoroughly discussing the related challenges. Additionally, prompt learning advances zero-shot learning, where task-specific prompts enable models trained on general datasets to tackle niche tasks without additional retraining. For instance, satellite imagery models can interpret prompts like “detect water bodies” to identify specific geographic features efficiently [149]. In Table 9, the key topics covered in this study concerning prompt learning are outlined.

### 3.9. Model Compression and Optimization Techniques for Efficiency and Scalability

As DL models become increasingly complex, model compression [150] and optimization techniques [151] have become essential for ensuring their efficiency and scalability, particularly in resource-constrained environments. Pruning is a key technique in model compression that reduces the size of a model by eliminating redundant or less significant parameters such as weights, neurons, or layers [152,153]. This not only decreases the model’s computational demands but also accelerates inference time and reduces memory usage, making it feasible to deploy DL models on edge devices with limited resources [154]. Advanced pruning strategies, such as those guided by RL, ensure that models retain their performance while becoming more efficient [155,156,157].

Quantization further contributes to optimization by reducing the precision of model parameters, converting them from 32-bit floating-point numbers to lower-bit representations, such as 8-bit integers [158,159]. This reduction significantly lowers the computational and memory requirements, enabling faster inference without compromising accuracy. Knowledge distillation is another powerful technique where a smaller, more efficient model (the student) learns from a larger, more accurate model (the teacher) [160]. This approach ensures that the student model retains the essential characteristics of the teacher while being optimized for deployment in real-time or resource-constrained environments. Together, these advanced optimization techniques are essential for extending the applicability of DL models across a wide range of real-world scenarios [161,162,163,164,165,166,167]. Finally, Table 10 categorizes the analyzed methods and highlights notable studies offering a reference for understanding the contributions of each technique to the field.

## 4. Advanced Deep Learning Models

The rapid progression of DL in image processing has been marked by the continuous development of advanced models that address the limitations of earlier architectures while introducing novel capabilities. These models represent the cutting edge of DL, incorporating sophisticated mechanisms that enable them to tackle increasingly complex and varied image processing tasks with greater accuracy, efficiency, and adaptability.

### 4.1. Deep Residual Networks and Beyond

ResNets are a seminal advancement in DL architecture, primarily addressing the degradation problem that arises when training deep NNs. As networks deepen, they often struggle with vanishing and exploding gradients, leading to deteriorated performance. ResNets tackle this issue by introducing skip connections, which allow the network to learn residual functions instead of directly mapping inputs to outputs. This approach enables the training of networks with hundreds of layers, significantly improving performance on complex image-processing tasks like classification and detection [168,169,170,171,172].

ResNets have inspired further innovations, such as ResNeXt and DenseNet, which expand on the concept of residual learning [173]. ResNeXt utilizes a split–transform–merge strategy to aggregate transformations, enhancing the model’s ability to capture diverse features, while DenseNet connects each layer to every other layer in a feed-forward manner, promoting feature reuse and improving efficiency. However, the depth and complexity of these models also introduce challenges [174,175].

### 4.2. Attention Mechanisms and Transformers

Attention mechanisms have revolutionized DL by enabling models to focus dynamically on the most relevant parts of the input data. Initially developed in the context of natural language processing, attention mechanisms have been adapted for image processing, where they enhance the ability to model complex spatial dependencies. Unlike traditional convolutional networks that apply fixed filters uniformly across the entire image, attention mechanisms weigh different regions according to their importance for the task at hand, significantly improving performance in tasks such as classification, object detection, and segmentation [176,177,178,179,180].

The introduction of ViTs marks a significant leap forward in leveraging attention mechanisms for image processing. ViTs treat images as sequences of patches, using self-attention to model long-range dependencies across the entire image [181]. This capability allows transformers to capture global context in a more flexible and scalable manner than traditional CNNs. However, transformers are computationally expensive, particularly as input sizes increase, posing challenges for real-time applications and deployments on edge devices. Future advancements may focus on hybrid models that combine the strengths of CNNs and transformers, offering a balance between local feature extraction and global context modeling [182,183,184,185].

### 4.3. Generative Models and Adversarial Networks

Generative models, especially GANs, have introduced new dimensions to DL in image processing. GANs consist of two NNs—the generator and the discriminator—engaged in a dynamic adversarial process where the generator creates synthetic images, and the discriminator attempts to distinguish between real and generated images. This interplay enables the generator to produce increasingly realistic images, making GANs highly effective for tasks such as image synthesis, style transfer, and super-resolution [186,187,188,189].

GANs have several limitations [190] that can hinder their application and performance. One major challenge is training instability, as the adversarial nature between the generator and discriminator often leads to convergence issues, making it difficult to achieve a balance between the two networks. Another common problem is mode collapse, where the generator produces limited or repetitive outputs instead of capturing the full diversity of the target data distribution. Techniques such as WGANs [191], which optimize a more stable loss function, and CGANs [192], which allow for controlled image generation based on auxiliary information, have been developed to address these issues. Despite the advancements, GANs require large and diverse datasets to perform effectively, limiting their applicability in data-scarce environments [193,194].

Also, GANs are highly sensitive to hyperparameter settings, requiring meticulous tuning of learning rates, batch sizes, and other factors to ensure optimal performance. Furthermore, GANs typically demand substantial computational resources, especially for tasks involving high-resolution images or complex data distributions. Moreover, GANs are prone to overfitting, where they memorize the training data instead of generalizing to new inputs, which can limit their effectiveness in real-world applications. These limitations highlight the need for careful design, training strategies, and evaluation methods when working with GANs. Lastly, evaluating GANs is also a significant challenge since there is no universally accepted metric to comprehensively assess their output quality and diversity [195].

### 4.4. Hybrid and Multi-Modal Models

Hybrid and multi-modal models represent a significant advancement in image processing, combining the strengths of different NN architectures or integrating diverse data modalities to enhance performance. For instance, hybrid models that integrate CNNs with transformers capture both spatial features in images and global dependencies, making them particularly effective for complex tasks such as video analysis or visual question answering [196,197,198,199,200,201].

In multi-modal settings, combining visual data with textual, auditory, or sensory inputs can significantly improve a model’s understanding and decision-making capabilities [202]. Effective fusion strategies, such as cross-modal transformers [203] and co-attentive networks [204], are critical for ensuring that combined data contribute meaningfully to the model’s performance. These advanced models are particularly impactful in applications such as medical diagnostics, where integrating imaging data with clinical records can lead to more accurate diagnoses, or in autonomous driving, where combining visual, LiDAR, and radar data enhances perception and decision-making processes [205,206].

Table 11 provides a comprehensive classification of the references discussed in this section, noting key advanced DL models across various categories and the contributions of each model type, offering insights into their unique functionalities and the specific challenges they address within the realm of image processing.

## 5. Evaluation Metrics for Image Processing Models

Evaluating the performance of DL models in image processing requires a set of well-defined and sophisticated metrics that accurately reflect the quality and efficacy of the models across different tasks. Given the diversity of tasks within image processing—ranging from classification and detection to segmentation and generation—each type of task necessitates specific metrics tailored to its unique requirements. In this section, we delve into the most critical evaluation metrics, providing rigorous mathematical definitions and equations, along with a discussion of their significance and application [207,208,209,210,211,212].

Accuracy is one of the most fundamental metrics for evaluating image classification tasks. It measures the proportion of correctly classified instances out of the total instances in the dataset. Mathematically, accuracy is defined as follows:

(1) $$\begin{array}{cc} \mathrm{Accuracy} & =\frac{\mathrm{Number}\;\mathrm{of}\;\mathrm{Correct}\;\mathrm{Predictions}}{\mathrm{Total}\;\mathrm{Number}\;\mathrm{of}\;\mathrm{Predictions}}\\ \; & =\frac{TP+TN}{TP+TN+FP+FN} \end{array}$$

where TP is the number of true positives, TN is the number of true negatives, FP is the number of false positives, and FN is the number of false negatives. Accuracy is most effective when the dataset is balanced; however, in cases of class imbalance, it may not provide a true reflection of model performance.

Precision and recall are crucial metrics for evaluating image processing tasks such as object detection and segmentation, where class imbalance is common. Precision measures the accuracy of positive predictions and is defined as follows:

(2) $$\mathrm{Precision}=\frac{TP}{TP+FP}.$$

Mean average precision (mAP) is widely used in object detection tasks to evaluate the precision–recall trade-off across different recall thresholds. mAP is calculated by averaging the average precision (AP) across all classes. The AP for each class is computed as follows:

(3) $$\mathrm{AP}=\int_{0}^{1}p\left(r\right)\;dr$$

where p(r) is the precision as a function of recall *r*. Then, mAP is given by

(4) $$\mathrm{mAP}=\frac{1}{N}\sum_{i=1}^{N}{\mathrm{AP}}_{i},$$

where *N* is the number of classes. mAP provides a comprehensive evaluation by considering both precision and recall across various thresholds.

Recall, also known as sensitivity or true positive rate, measures the ability of the model to correctly identify all positive instances and is defined as follows:

(5) $$\mathrm{Recall}=\frac{TP}{TP+FN},$$

Both precision and recall are critical in contexts where the cost of false positives or false negatives is high.

The F1-Score is the harmonic mean of precision and recall, providing a single metric that balances the trade-off between the two. It is particularly useful when the distribution of classes is uneven and a balance between precision and recall is desired. The F1-Score ranges from 0 to 1, with 1 indicating perfect precision and recall, and is defined as follows:

(6) $$F1-\mathrm{Score}=2\times \frac{\mathrm{Precision}\times \mathrm{Recall}}{\mathrm{Precision}+\mathrm{Recall}}=\frac{2TP}{2TP+FP+FN}.$$

Intersection over Union (IoU) is a critical metric for evaluating object detection and segmentation tasks. Mathematically, IoU is defined as follows:

(7) $$\begin{array}{cc} \mathrm{IoU} & =\frac{\mathrm{Area}\;\mathrm{of}\;\mathrm{Overlap}}{\mathrm{Area}\;\mathrm{of}\;\mathrm{Union}}\\ \; & =\frac{\left|A\cap B\right|}{\left|A\cup B\right|}, \end{array}$$

where the “Area of Overlap” represents the region where the predicted result and the ground truth agree, while the “Area of Union” captures the total area covered by both the predicted result and the ground truth. More specifically, *A* is the predicted bounding box or segmentation mask, and *B* is the ground truth bounding box or segmentation mask. An alternative definition of IoU is

(8) $$\mathrm{IoU}=\frac{TP}{TP+FP+FN},$$

where TP denotes the region correctly predicted as part of the object, FP captures the region predicted as part of the object but is not part of the ground truth, and FN represents the region that belongs to the object in the ground truth but is not captured by the prediction. IoU measures how well the predicted region overlaps with the actual ground truth. IoU values range from 0 to 1, with higher values indicating better model performance. An IoU threshold (e.g., 0.5) is often used to determine whether a detection is considered a true positive. A perfect IoU (IoU = 1) score means that the predicted region perfectly matches the ground truth (no false positives or false negatives). Lower IoU scores (IoU < 1) indicate that there is either over-prediction (false positives) or under-prediction (false negatives).

The Jaccard Index, also known as the Jaccard Similarity Coefficient, is another metric used for segmentation tasks. It is often referred to separately in the context of binary segmentation. The Jaccard Index, like IoU, provides a measure of similarity between the predicted and ground truth masks, with values ranging from 0 (no overlap) to 1 (perfect overlap). The IoU and Jaccard Index are mathematically identical metrics used to measure the similarity between predicted and ground truth regions, particularly in segmentation tasks. The Jaccard Index originates from set theory as a general measure of set similarity, while IoU is a term more commonly used in computer vision, specifically for tasks like object detection and segmentation. In binary segmentation, the terms are often used interchangeably, but the Jaccard Index is sometimes highlighted separately to emphasize its historical roots and application in simple binary tasks. In contrast, IoU is more widely associated with multi-class scenarios, where mean IoU (average IoU across all classes) is often discussed, and performance thresholds, such as in object detection. Despite these contextual differences, they provide identical numerical evaluations of overlap quality.

The Dice Coefficient is a metric similar to IoU but is specifically tailored for evaluating segmentation tasks (emphasizing the overlap more strongly). It measures the overlap between two samples and is particularly useful in scenarios where the target object occupies a small area in the image. While IoU is widely used in general computer vision tasks, the Dice Coefficient is often favoured in applications like medical imaging due to its sensitivity to smaller regions. Both provide useful insights into the quality of segmentation models. The Dice Coefficient is defined as follows:

(9) $$\mathrm{Dice}\;\mathrm{Coefficient}=\frac{2\times |A\cap B|}{\left|A|+|B\right|},$$

where *A* is the predicted segmentation mask, and *B* is the ground truth segmentation mask. The Dice Coefficient quantifies the degree of similarity by comparing the size of the overlap (True Positives) to the combined size of the predicted and actual regions. The formula is given by

(10) $$\mathrm{Dice}\;\mathrm{Coefficient}=\frac{2TP}{2TP+FP+FN},$$

where TP are pixels correctly identified as part of the target object, FP the pixels incorrectly identified as belonging to the object, and FN are pixels that belong to the object but were missed in the prediction. A Dice Coefficient of 1 indicates perfect agreement between the prediction and ground truth, while 0 represents no overlap. This metric is particularly effective in segmentation tasks where precise boundary matching is crucial, such as in medical imaging or autonomous systems.

Pixel accuracy is a straightforward metric used in segmentation tasks. It measures the proportion of correctly classified pixels over the total number of pixels in the image:

(11) $$\mathrm{Pixel}\;\mathrm{Accuracy}=\frac{\sum_{i=1}^{N}I\left({\hat{y}}_{i}=y_{i}\right)}{N},$$

where *N* is the total number of pixels, ${\hat{y}}_{i}$ is the predicted label for pixel *i*, and $y_{i}$ is the ground truth label for pixel *i*. While easy to compute, pixel accuracy may not be sufficient in cases where the classes are imbalanced, as it could overestimate the performance by ignoring small but critical regions.

The Structural Similarity Index (SSIM) is a perceptual metric that quantifies the similarity between two images. Unlike traditional metrics that measure absolute errors, SSIM takes into account changes in structural information, luminance, and contrast. It is defined as follows:

(12) $$\mathrm{SSIM}\left(x,y\right)=\frac{\left(2\mu_{x}\mu_{y}+C_{1}\right)\left(2\sigma_{xy}+C_{2}\right)}{\left(\mu_{x}^{2}+\mu_{y}^{2}+C_{1}\right)\left(\sigma_{x}^{2}+\sigma_{y}^{2}+C_{2}\right)},$$

where *x* and *y* are the two images being compared, $\mu_{x}$ and $\mu_{y}$ are the mean intensities of *x* and *y*, $\sigma_{x}^{2}$ and $\sigma_{y}^{2}$ are the variances of *x* and *y*, $\sigma_{xy}$ is the covariance of *x* and *y*, and $C_{1}$ and $C_{2}$ are constants to avoid division by zero. SSIM values range from −1 to 1, with higher values indicating greater structural similarity.

The Fréchet Inception Distance (FID) is a metric used to evaluate the quality of images generated by models like GANs. FID compares the distribution of generated images with that of real images using the features extracted from a pre-trained network, typically the Inception model. It is defined as follows:

(13) $$\mathrm{FID}=∥\mu_{r}-\mu_{g}{∥}^{2}+\mathrm{Tr}\left(\Sigma_{r}+\Sigma_{g}-2{\left(\Sigma_{r}\Sigma_{g}\right)}^{1/2}\right),$$

where $\mu_{r}$ and $\mu_{g}$ are the mean feature vectors for the real and generated images, respectively, and $\Sigma_{r}$ and $\Sigma_{g}$ are the covariance matrices for the real and generated images, respectively. Lower FID values indicate that the generated images are more similar to the real images, with values closer to zero being ideal.

The peak signal-to-noise ratio (PSNR) is a metric used to measure the quality of reconstruction in tasks like image super-resolution and compression. It compares the maximum possible signal to the noise affecting the fidelity of its representation, calculated as follows:

(14) $$\mathrm{PSNR}=10\cdot \log_{10}\left(\frac{{\mathrm{MAX}}_{I}^{2}}{\mathrm{MSE}}\right),$$

where ${\mathrm{MAX}}_{I}$ is the maximum possible pixel value of the image (e.g., 255 for an 8-bit image), and MSE is the mean squared error between the original and reconstructed images. Higher PSNR values indicate better reconstruction quality.

Normalized Cross-Correlation (NCC) is used in template matching and registration tasks, measuring the similarity between two images. It is defined as follows:

(15) $$\mathrm{NCC}=\frac{\sum_{i}\left(I_{i}-\bar{I}\right)\left(T_{i}-\bar{T}\right)}{\sqrt{\sum_{i}{\left(I_{i}-\bar{I}\right)}^{2}\sum_{i}{\left(T_{i}-\bar{T}\right)}^{2}}},$$

where $I_{i}$ and $T_{i}$ are the intensity values of the image and template, respectively, and $\bar{I}$ and $\bar{T}$ are the mean intensities of the image and template. NCC values range from −1 to 1, where 1 indicates perfect correlation.

Cohen’s Kappa is a statistical measure of inter-rater agreement or reliability, often used in classification tasks to assess the agreement between predicted and true classifications beyond chance. It is defined as follows:

(16) $$\kappa =\frac{p_{o}-p_{e}}{1-p_{e}},$$

where $p_{o}$ is the observed agreement, and $p_{e}$ is the expected agreement by chance. Cohen’s Kappa ranges from −1 to 1, with 1 indicating perfect agreement and values less than 0 indicating agreement worse than chance.

The Receiver Operating Characteristic (ROC) curve is a graphical plot that illustrates the diagnostic ability of a binary classifier system by varying its discrimination threshold. The Area Under the ROC Curve (AUC) provides a single scalar value to summarize the overall performance of the classifier:

(17) $$\mathrm{AUC}=\int_{0}^{1}TPR\left(FPR\right)\;d\left(FPR\right),$$

where TPR is the true positive rate, and FPR is the false positive rate. AUC values range from 0 to 1, with values closer to 1 indicating better model performance.

Logarithmic Loss, or Log Loss, measures the performance of a classification model where the prediction is a probability value between 0 and 1. The log loss increases as the predicted probability diverges from the actual label:

(18) $$\mathrm{Log}\;\mathrm{Loss}=-\frac{1}{N}\sum_{i=1}^{N}\left[y_{i}\log\left(p_{i}\right)+\left(1-y_{i}\right)\log\left(1-p_{i}\right)\right],$$

where *N* is the number of instances, $y_{i}$ is the actual label (0 or 1), and $p_{i}$ is the predicted probability of the instance being in class 1. Lower Log Loss values indicate better performance.

Lastly, Balanced Accuracy and Matthews Correlation Coefficient (MCC) are advanced metrics used in cases of imbalanced datasets. Balanced accuracy is the average of recall obtained in each class:

(19) $$\mathrm{Balanced}\;\mathrm{Accuracy}=\frac{1}{2}\left(\frac{TP}{TP+FN}+\frac{TN}{TN+FP}\right),$$

MCC provides a comprehensive metric that considers all four quadrants of the confusion matrix:

(20) $$\mathrm{MCC}=\frac{TP\times TN-FP\times FN}{\sqrt{\left(TP+FP)(TP+FN)(TN+FP)(TN+FN\right)}}.$$

MCC ranges from −1 to 1, where 1 indicates perfect prediction, 0 indicates no better than random prediction, and −1 indicates total disagreement between predictions and actual outcomes.

The choice of evaluation metrics is crucial for accurately assessing the performance of DL models in image processing. Each metric provides unique insights into different aspects of model performance, from accuracy and precision to structural similarity and generation quality.

Table 12 provides an overview of the evaluation metrics discussed in this section, categorizing them based on their application to different tasks in image processing. These metrics play a crucial role in assessing the performance and reliability of DL models in diverse scenarios. For instance, classification metrics such as accuracy, precision, recall, and F1-Score are widely used in tasks like object recognition and disease classification, where the balance between false positives and false negatives is critical. In segmentation tasks, metrics like IoU and Dice Coefficient are essential for evaluating the overlap between predicted and ground truth masks, particularly in medical imaging applications, such as tumor detection, where precise boundaries are crucial. For image quality assessment, metrics such as SSIM and PSNR are ideal for evaluating reconstruction tasks, such as super-resolution or denoising, where perceptual similarity matters. In object detection, metrics like mAP are commonly used to evaluate how well models identify and localize objects in scenes, as seen in autonomous driving systems. Lastly, advanced evaluation metrics like FID are indispensable for assessing the realism of generated images in applications involving generative models. By categorizing these metrics and providing practical guidance, Table 12 serves as a source for selecting the most appropriate evaluation metrics for specific image processing tasks.

## 6. Applications of Deep Learning in Image Processing

DL has profoundly impacted a wide array of domains through its ability to process and interpret complex visual data. Its applications span numerous fields, from healthcare to autonomous systems, each benefiting from the unique capabilities of DL models. This section explores some of the most significant and transformative applications, demonstrated in Table 13, highlighting recent advancements, ethical considerations, and interdisciplinary collaborations.

### 6.1. Medical Imaging

Medical imaging has been one of the most impactful areas for the application of DL in image processing. CNNs have revolutionized diagnostic processes, enabling the detection of diseases such as cancer, Alzheimer’s, and diabetic retinopathy with remarkable accuracy. For instance, DL models have been developed to identify early-stage tumours in mammograms that might be missed by the human eye. These advancements extend beyond diagnosis to treatment planning and monitoring, where models assist in delineating tumours in radiotherapy and predicting patient outcomes [213,214,215,216,217,218,219,220,221].

Recent innovations, such as self-supervised learning, are further enhancing the field, enabling models to learn from vast amounts of unlabeled medical images, which are often more abundant than labeled data. Additionally, there is a growing integration of AI with wearable technology, facilitating continuous monitoring and early detection of health issues. However, these advancements also bring challenges, particularly in terms of bias in training data that can lead to disparities in diagnostic accuracy across different demographic groups. Addressing these biases, ensuring model interpretability, and adhering to stringent regulatory standards are crucial for the responsible deployment of AI in healthcare [222,223,224,225,226,227,228].

### 6.2. Autonomous Systems

Autonomous vehicles rely heavily on DL for a variety of tasks, including object detection, lane keeping, and obstacle avoidance. The ability of DL models to process real-time video data and make split-second decisions is critical for the safe operation of these vehicles. For example, DL is central to the functioning of advanced driver-assistance systems (ADASs) in vehicles from various companies, where models must accurately detect and respond to pedestrians, other vehicles, and road signs under varying environmental conditions [229,230,231,232,233,234,235].

Recent developments in real-time AI and edge computing have further improved the efficiency and reliability of autonomous systems. By processing data closer to the source, edge computing reduces latency and enables faster decision-making, which is crucial in dynamic driving environments. However, significant challenges remain, particularly in ensuring that models generalize well across diverse and unpredictable driving scenarios. Collaboration between AI researchers, automotive engineers, and policymakers is essential to address these challenges and advance the field [236,237,238,239,240].

### 6.3. Remote Sensing and Environmental Monitoring

Remote sensing and environmental monitoring have also greatly benefited from DL, particularly in the analysis of satellite and aerial imagery. DL models are used to monitor deforestation, track wildlife, assess damage from natural disasters, and predict crop yields. For instance, during disaster response, these models can quickly analyze satellite images to assess the extent of damage and identify areas in need of immediate aid [241,242,243,244,245,246,247].

The integration of DL with remote sensing has enabled more accurate and timely decision-making, which is crucial in managing environmental challenges and responding to natural disasters. Moreover, the advent of self-supervised and semi-supervised learning techniques is allowing models to better handle the vast amounts of unlabeled data typical in this field. However, the computational cost of processing high-resolution satellite images remains a challenge, and there is ongoing research into making these models more efficient and scalable [248,249,250,251,252,253].

### 6.4. Security and Surveillance

Security and surveillance is another domain where DL is making significant strides. From facial recognition systems to automated threat detection in public spaces, DL models are increasingly being deployed to enhance security. These systems can analyze vast amounts of video data in real time, identifying potential threats and reducing the burden on human operators [254,255,256,257,258,259,260].

However, the deployment of DL in surveillance raises serious ethical concerns, particularly regarding privacy and civil liberties. The potential for misuse of facial recognition technology by governments or corporations, as well as the risk of bias in these systems, which could lead to discriminatory practices, are significant challenges that need to be addressed. Research is focused on developing privacy-preserving algorithms and ensuring that these technologies are used in a manner that respects individual rights and freedoms [261,262,263,264,265,266,267].

### 6.5. Art and Cultural Heritage

Art and cultural heritage preservation is a more unconventional but equally important application of DL. Models are being used to restore damaged artworks, colorize black-and-white photographs, and even generate new art in the style of famous artists. DL is also helping to digitize and analyze vast collections of cultural artifacts, making them more accessible to the public and preserving them for future generations [268,269,270,271,272,273].

In this domain, the focus is not only on technological advancement but also on interdisciplinary collaboration. Art historians, conservators, and AI researchers are working together to ensure that the application of DL respects the integrity and cultural significance of the artifacts. Additionally, there is a growing interest in using AI to enhance the public’s engagement with art and culture through interactive and immersive experiences [274,275,276,277,278].

### 6.6. Ethical and Social Considerations

Across all these applications, ethical and social considerations are paramount. The deployment of DL technologies raises important questions about privacy, bias, and fairness. For example, in the context of surveillance and security, there is a significant risk that these technologies could infringe on individual privacy or be used in ways that exacerbate social inequalities. Similarly, in medical imaging, bias in training data can lead to disparities in diagnosis and treatment outcomes across different demographic groups [279,280,281,282,283,284,285].

To address these concerns, it is crucial to develop frameworks and standards that ensure the responsible use of AI. This includes implementing privacy-preserving techniques, designing algorithms that are fair and unbiased, and ensuring transparency and accountability in AI systems. The ethical deployment of DL technologies requires a careful balance between innovation and the protection of fundamental human rights [286,287,288,289,290,291,292].

### 6.7. Interdisciplinary Collaboration

The successful application of DL in image processing often requires interdisciplinary collaboration. In many of the domains discussed, the most impactful advancements have come from partnerships between experts in computer science, domain-specific fields (such as medicine or environmental science), ethics, and law. For instance, in healthcare, the collaboration between AI researchers and clinicians is crucial for developing models that are not only accurate but also clinically relevant and ethically sound [293,294,295,296,297,298].

Interdisciplinary collaboration ensures that the application of DL is informed by a deep understanding of the context in which it is deployed, leading to more effective and responsible AI solutions. By bringing together diverse perspectives and expertise, these collaborations can help to address complex challenges and maximize the benefits of DL across various domains [299,300,301,302,303,304].

## 7. Challenges and Future Directions

As DL continues to revolutionize the field of image processing, it faces several significant challenges that must be addressed to ensure the development of robust, scalable, and ethically sound models. These challenges also open up avenues for future research and innovation, as the field evolves to meet the growing demands of various applications.

### 7.1. Challenges

One of the foremost challenges in DL for image processing is data scarcity, particularly in specialized domains such as medical imaging, autonomous vehicles, and satellite imagery. In these areas, obtaining large, annotated datasets is not only difficult but also costly, requiring expert knowledge for accurate labeling. This scarcity hinders the training of DL models, which typically require vast amounts of data to achieve high performance. Although techniques like data augmentation and synthetic data generation have been employed to mitigate this issue, they often fall short of providing the diversity and realism needed for truly effective model training [305,306,307,308,309,310].

Another critical challenge is the computational complexity of DL models. As models grow in size and complexity, they demand significant computational resources for both training and inference. This becomes a major hurdle when deploying models on edge devices or in real-time applications where computational power is limited. Furthermore, the energy consumption of large-scale models is increasingly becoming a concern, particularly in the context of sustainable AI practices [311,312,313,314,315,316].

Interpretability remains a significant barrier to the widespread adoption of DL in critical fields such as healthcare, finance, and law. The “black-box” nature of many DL models means that their decision-making processes are often opaque, making it difficult to trust and validate their outputs. This lack of transparency can lead to resistance from stakeholders and regulatory bodies, who require clear justifications for the decisions made by AI systems. The challenge here is not only to develop more interpretable models but also to balance interpretability with performance, as increasing one often comes at the expense of the other [317,318,319,320,321,322].

Generalization and robustness are also ongoing challenges in DL. Models that perform exceptionally well on training data often struggle to maintain that performance on unseen data, particularly when there is a shift in the data distribution or when the models are exposed to adversarial examples. Ensuring that models generalize well across different environments and are robust to variations and attacks is critical for their reliable deployment in real-world applications [323,324,325,326,327,328].

Lastly, the ethical implications of deploying DL models in image processing applications cannot be overlooked. Bias in training data can lead to models that reinforce or exacerbate existing societal inequalities, particularly in applications like facial recognition and predictive policing. Privacy concerns arise when AI is used in surveillance or other contexts where sensitive personal information is processed. Addressing these ethical challenges requires a concerted effort to develop fair, transparent, and accountable AI systems [329,330,331,332,333,334].

A detailed overview of the primary challenges discussed in this section is provided in Table 14, encompassing data scarcity, computational complexity, interpretability, generalization, and ethical considerations. These challenges represent critical hurdles in the development and deployment of effective DL models for image processing, as they impact the scalability, reliability, and transparency of these technologies. Finally, the table synthesizes key references, offering a structured foundation to understand the scope and implications of each challenge within this rapidly evolving field.

### 7.2. Future Directions

To address these challenges, several promising directions for future research and development have emerged. One of the most significant is the advancement of self-supervised learning techniques. By leveraging vast amounts of unlabeled data, self-supervised learning can help alleviate the issue of data scarcity, allowing models to learn useful representations without the need for extensive labeled datasets. This approach not only reduces the reliance on labeled data but also enhances the model’s ability to generalize across different tasks and domains [335,336,337,338,339,340].

The development of more efficient model architectures is another critical area of focus. Innovations such as neural architecture search (NAS), pruning, quantization, and distillation are driving the creation of models that are both powerful and computationally efficient. These techniques enable the deployment of DL models on edge devices and in real-time applications, broadening the accessibility and applicability of AI. Additionally, exploring new hardware paradigms, such as neuromorphic and quantum computing, could further revolutionize how DL models are designed and deployed [341,342,343,344,345,346].

Explainable AI (XAI) is becoming increasingly important as we seek to build trust in AI systems. Research into methods that can make DL models more interpretable without sacrificing performance is gaining momentum. Techniques such as attention mechanisms, feature attribution methods, and interpretable model architectures are crucial for creating AI systems that are transparent and trustworthy. Additionally, developing standards for AI explainability and integrating them into regulatory frameworks will be essential for the broader adoption of AI in sensitive fields [347,348,349,350,351,352,353].

Another exciting direction is the integration of emerging technologies with DL. Quantum computing, for example, holds the potential to exponentially accelerate certain computations, making it possible to train and deploy much larger and more complex models. Edge computing, which brings computation closer to the data source, could revolutionize real-time image-processing tasks by reducing latency and improving privacy. The convergence of these technologies with DL could lead to groundbreaking innovations in areas such as autonomous vehicles, smart cities, and personalized medicine [354,355,356,357,358,359].

Finally, as AI becomes increasingly pervasive, there is a growing need to develop new evaluation metrics that go beyond traditional accuracy and performance measures. These metrics should capture aspects such as robustness, fairness, and ethical considerations, ensuring that models are not only technically sound but also socially responsible. The development of such metrics, along with frameworks for continuous monitoring and auditing of AI systems, will be crucial for ensuring that AI technologies are aligned with societal values [360,361,362,363,364,365].

In summary, Table 15 outlines the promising future directions explored in this section. This includes advancements in self-supervised learning, efficient model architectures, explainable AI, integration with emerging technologies, and the development of new evaluation metrics. While the challenges facing DL in image processing are significant, they also present opportunities for innovation. By advancing research in the aforementioned directions, the field can continue to evolve, addressing the limitations of current approaches and opening up new possibilities for the future [366,367,368,369,370,371,372].

## 8. Conclusions

DL has fundamentally transformed the landscape of image processing, driving unprecedented advancements across various domains. This survey has provided a comprehensive examination of the key models, techniques, and evaluation metrics that have propelled DL to the forefront of image processing research and application. By tracing the evolution of DL architectures from their inception to the latest state-of-the-art models, we have highlighted the critical innovations that have enabled these models to achieve remarkable success in handling complex visual data.

This survey has underscored the importance of advanced techniques that enhance model performance, such as automated feature extraction, transfer learning, and attention mechanisms. These techniques have not only improved the accuracy and generalization capabilities of DL models but have also expanded their applicability to a wide range of image processing tasks, from basic image recognition to sophisticated tasks like semantic segmentation and image generation.

Furthermore, we have explored the metrics used to evaluate these models, emphasizing the need for rigorous and context-specific assessment to ensure that DL models meet the high standards required for real-world deployment. The discussion on evaluation metrics highlights the nuanced understanding needed to interpret model performance accurately, particularly in diverse and challenging application scenarios.

This survey has also identified the persistent challenges that continue to hinder the full potential of DL in image processing. Issues such as data scarcity, high computational costs, and the black-box nature of DL models present significant obstacles that must be addressed to further advance the field. These challenges underscore the importance of ongoing research into more efficient, interpretable, and accessible DL methodologies.

Looking forward, the integration of DL with emerging technologies such as edge computing, quantum computing, and self-supervised learning offers exciting possibilities for the future of image processing. These advancements have the potential to overcome current limitations, enabling more efficient, scalable, and interpretable models that can be deployed across a wider array of applications, even in resource-constrained environments.

While this survey offers a comprehensive overview of DL techniques and models in image processing, it has several limitations that should be acknowledged. This study primarily focuses on established and recent advancements, potentially under-representing the latest breakthroughs and emerging technologies, such as quantum computing and neuromorphic architectures. Additionally, this survey does not provide in-depth comparative analyses between models under consistent evaluation metrics, limiting practical insights. Interdisciplinary considerations and the role of collaboration in addressing real-world challenges are briefly discussed. Furthermore, while ethical and social implications, such as biases and privacy concerns, are mentioned, they are not explored in depth. These limitations highlight areas for further research, including a more detailed exploration of emerging trends, domain-specific applications, and ethical challenges in deploying DL models.

In summary, this survey not only provides a synthesis of the current state of DL in image processing but also offers a forward-looking perspective on the future directions of the field. By consolidating the vast and diverse body of research into a cohesive overview, this survey serves as a valuable resource for both researchers and practitioners. It lays the groundwork for future innovations, guiding the continued evolution of DL as a transformative force in image processing. The insights presented here aim to inspire further exploration and development, ensuring that DL remains at the cutting edge of image processing technology.

## Figures and Tables

**Table 1:** Summary of architectural innovations in DL for image processing.

Architecture	Innovation	References
CNNs	They are the foundation for image processing, enable automatic spatial hierarchy capture through convolutional layers, which process image patterns at different levels of granularity.	[19]
ResNets	Introduced residual connections to address vanishing gradient problems, allowing deeper networks to be trained by learning residual functions rather than direct mappings.	[20,21]
DenseNets	DenseNets enable direct connections between all layers to enhance feature reuse, reduce computational costs, and improve efficiency in image classification and object detection tasks.	[22,23,24]
Multi-branch Architectures	Inception networks process image features at multiple scales simultaneously within a single model, significantly improving performance on complex tasks like semantic segmentation.	[25,26,27,28]
YOLO	YOLO transformed object detection with a single network approach, simultaneously predicting bounding boxes and class probabilities, achieving real-time efficiency with high accuracy.	[29,30]
ConvNext	ConvNext integrates principles from vision transformers into traditional CNNs, improving performance with innovations like depth-wise convolutions and larger kernel sizes while retaining simplicity.	[31,32,33]
FCNs	FCNs replace fully connected layers with convolutional ones, preserving spatial hierarchies for dense predictions in tasks such as semantic segmentation.	[34]
U-Net	U-Net’s encoder–decoder structure with skip connections enables precise boundary delineation, making it especially effective for medical imaging and other pixel-level prediction tasks.	[35,36,37]
Mask R-CNN	Mask R-CNN extends object detection by integrating segmentation, creating pixel-level masks for detected objects, which is valuable for tasks like autonomous driving and video analysis.	[38,39]
Specialized Task-Specific Architectures	Tailored architectures address specific challenges in advanced image processing, ensuring accuracy and efficiency in highly specialized domains.	[40,41,42]
ViTs	Vision transformers handle global dependencies in images by modeling them as sequences of patches, offering advantages in scene understanding and holistic image analysis	[43,44,45,46,47]
Self-Attention Mechanisms	Self-attention dynamically prioritizes relevant image regions for tasks like classification and segmentation, enabling robust generalization across diverse datasets.	[48,49,50,51,52,53]
GANs	GANs use adversarial training between a generator and discriminator to create realistic images, excelling in tasks like image synthesis, super-resolution, and style transfer.	[54,55,56,57]
CGANs	CGANs integrate class labels or other auxiliary information into GANs, enabling controlled generation of specific types of images based on given conditions.	[58]
WGANs	WGANs improve GAN training stability by introducing a Wasserstein distance-based loss function, addressing mode collapse and convergence issues.	[59]
Other GAN Applications	GANs are used for synthetic data generation, data augmentation, and domain adaptation, improving robustness and generalization in low-data scenarios and cross-domain tasks.	[60,61,62,63,64]
Diffusion Models	Diffusion models utilize a probabilistic framework to iteratively add and remove noise, achieving state-of-the-art results in tasks like image restoration, synthesis, and denoising.	[65,66]

**Table 2:** Summary of topics in the context of transfer learning.

Topic	Description	References
Pre-trained models and transfer learning strategies	Analyze transfer learning using CNN- and transformer-based pre-trained models and their application in medical imaging. Also focused on key categories, i.e., adversarial-based and network-based (fine-tuning, freezing CNN layers, and progressive learning).	[67,68,69,70,71,72,73]
Negative transfer	Highlights the issue of negative transfer, where source and target tasks differ significantly, hindering performance, and strategies to mitigate its impact.	[74,75]
Negative transfer: mitigation strategies	Explores data transferability, model transferability, training process enhancement, and prediction refinement strategies.	[76]
	Concept-wise fine-tuning is a model transferability method.	[77]
	HTPL, a feature-based transfer learning approach that progressively fine-tunes features ensuring effective domain alignment and mitigating negative transfer issues.	[78]

**Table 3:** Summary of data augmentation techniques.

Technique	Description	References
Basic Methods	Basic augmentation techniques (e.g., rotation, scaling) for increasing dataset diversity and preventing overfitting, especially when large datasets are impractical or expensive.	[79,80,81,82,83]
Advanced Modern Approaches	Techniques like Cutout, Mixup, and CutMix enhance model robustness by introducing complex image variations and encouraging focus on global context rather than localized features.	[84]
Complex Image Transformations	Reviews the application of techniques such as blending images, masking, and targeted transformations to improve model generalization and reduce overfitting.	[85,86,87]
Automated Strategies	AutoAugment and RandAugment that leverage optimization and RL to identify the most effective augmentation policies for specific datasets, significantly improving performance with reduced manual effort.	[88,89,90]

**Table 4:** Summary of regularization topics and techniques.

Topic/Technique	Description	References
Comprehensive survey in Regularization	Reviews traditional and modern regularization methods, comparing their effectiveness, computational cost, and applicability to mitigate overfitting in DL.	[91]
Dropout	Prevents overfitting by randomly deactivating neurons during training, forcing the model to learn redundant feature representations and increasing robustness.	[92,93,94]
Weight Decay	Penalizes large weights by adding a regularization term to the loss function, discouraging excessive reliance on specific parameters and improving generalization. Also, disharmony issues are discussed with weight normalization methods.	[95,96]
Batch Normalization	Stabilizes and accelerates training by normalizing layer inputs, reducing internal covariate shift, and indirectly functioning as a regularization technique to improve model performance.	[97,98]

**Table 5:** Summary of aspects in adversarial training.

Topic	Description	References
Adversarial examples and Training	Overview of methods, challenges, and opportunities for generating adversarial examples to expose and improve model robustness.	[99,100,101,102,103]
FGSM	A computationally efficient method for generating adversarial examples with minimal perturbation.	[104]
PGD	An iterative approach and stronger method for crafting adversarial examples by refining perturbations stepwise.	[105]
Free Adversarial Training	Efficiently reuses gradient computations via minibatch replays to achieve robustness with reduced cost.	[106]
UPGD	Enhanced PGD algorithm for generating universal adversarial perturbations, balancing accuracy and robustness.	[107]
MAT	Leverages models of natural variation to generate adversarial examples, enhancing robustness against naturally shifted datasets.	[108]

**Table 6:** Summary of self-supervised and unsupervised learning techniques.

Technique	Description	References
Self-supervised and Unsupervised Learning	Techniques focusing on how they improve image classification without labeled data.	[109]
Proxy-based Learning	Frameworks for spectral–spatial hyperspectral image classification, enhancing robustness and efficiency.	[110]
Contrastive Learning	Reviews contrastive learning techniques in self-supervised frameworks, highlighting their success in extracting meaningful representations.	[111]
Multi-task Learning	Proposes frameworks for self-supervised learning in specific domains like medical imaging.	[112]
Self-supervised Learning Improvements	Mitigating issues related to proxy task specificity, improving performance across various downstream applications.	[113]
Representation Learning	Explores the combinations of labeled and unlabeled data for unified speech and visual representation.	[114]
	Reviews advances and challenges in self-supervised representation learning, emphasizing its potential in scalable applications.	[115]
	Context Autoencoders: demonstrates their use for effective representation learning in image processing tasks.	[116]
Self-supervised methods	Reviews key methods such as SimCLR and MoCo, focusing on robust representation learning through contrastive approaches.	[117,118,119]
Advanced self-supervised techniques	Integration of attention mechanisms and other advanced approaches to enhance capabilities in self-supervised frameworks.	[120,121,122]
Unsupervised Learning and Dimensionality reduction	Examines the use of clustering with UMAP in uncovering intrinsic structures of data, improving latent feature representations. Highlights the effectiveness of preserving essential features while reducing redundancy.	[123,124]
3D Convolutional Autoencoders	Explains their application in compact representation of hyperspectral image data.	[125]
Unsupervised Learning frameworks	Discusses applications into tasks such as medical image analysis, denoising, and segmentation.	[126,127,128]

**Table 7:** Summary of domain generalization and adaptation techniques.

Technique	Description	References
Domain variability	Discusses the challenges caused by domain shifts, such as differences in lighting, resolution, or imaging devices.	[129]
Domain generalization	Focuses on training models to perform robustly on unseen domains without direct access to target domain data during training.	[130]
DDC	Aligns feature distributions across multiple source domains for robust generalization.	[131]
DICA	Aligns features for learning domain-invariant representations.	[132]
Episodic training	Frameworks to enhance robustness by simulating domain shifts during training to prepare models for unseen variations.	[133]
DANNs	Adversarial learning to generate domain-agnostic features, improving generalization across domains.	[134]
CycleGAN	Transform target domain images into the appearance of the source domain (style transfer), improving alignment.	[135]
	Demonstrates its application in autonomous driving for adapting object detection models in rural environments despite training in urban areas.	[136]

**Table 8:** Summary of meta-learning categories and methods.

Technique	Description	References
Metric-based	Prototypical, siamese, and matching networks classify new data points by comparing them to learned class prototypes using feature extractors and similarity metrics.	[137,138]
Model-based	MANNs combine NNs with external memory modules to enhance learning efficiency. The SNAIL improves parameter tuning efficiency.	[139]
Optimization-based	MAML fine-tunes model parameters for rapid task adaptation. Other methods: META-LSTM, META-SGD, Reptile.	[138,139,140]

**Table 9:** Summary of prompt learning techniques.

Technique	Description	References
Vision-language models (e.g., CLIP)	Enables classification tasks using textual prompts enhancing applications like environmental monitoring and disaster assessment.	[141,142,143,144]
Interactive segmentation	Click prompt learning for real-time refinement of outputs using user-provided prompts particularly useful in medical imaging. Interactive Medical Image Learning Framework using DL algorithms trained during the user study compared in performance against state-of-the-art modern augmentations. PRISM model applied to 3D medical images’ segmentation with significantly improved performance. In-depth analysis of the foundational principles of interactive segmentation methodologies and categorization based on common characteristics in the field of medical imaging.	[145,146,147,148]
Zero-shot learning	Utilizes task-specific prompts to adapt models trained on general datasets for niche tasks, such as land-use mapping or satellite imagery analysis without retraining.	[149]

**Table 10:** Summary of model compression and optimization techniques.

Technique	Description	References
Model Compression Overview	Highlights the need for compression in DL to reduce computational cost and memory usage for deployment in resource-constrained environments such as edge devices.	[150]
Pruning	Removing redundancy or insignificant parameters (e.g., weights, neurons, layers) to reduce the model size, computational requirements, and inference time. Includes structured and unstructured pruning approaches.	[151,152,153,154,155,156,157]
Quantization	Reduced parameter precision (e.g., from 32-bit floats to 8-bit integers) for faster computation and memory usage while preserving model accuracy, enabling efficient deployment.	[158,159]
Knowledge Distillation	Transfers knowledge from a large teacher model to a smaller student model, preserving essential characteristics while enhancing scalability and efficiency for deployment.	[160,161]
Energy-Efficient Architectures	Focuses on developing architectures that consume less power and are optimized for specific hardware, including FPGA and ASIC implementations.	[162]
Self-Distillation Methods	Refine models using predictions from their intermediate layers as supervisory signals, improving performance without external teacher models.	[163,164]
RL for Optimization	Explores RL-based strategies to automate pruning and quantization, achieving optimal compression and performance trade-offs.	[165,166]
Cooperative Compression	Discusses collaborative approaches like joint optimization of pruning, quantization, and distillation for maximum resource utilization and scalability.	[167]

**Table 11:** Summary of advanced DL models.

Model	Description	References
ResNets	Introduced skip connections to address vanishing gradient problems, enabling the training of very deep networks and improving performance in tasks like classification and detection.	[168,169,170,171,172]
ResNeXt	Utilized a split–transform–merge strategy to aggregate transformations, enhancing feature diversity and capture efficiency.	[173]
DenseNet	Introduced densely connected layers to promote feature reuse, reduce the number of parameters, and improve computational efficiency and accuracy.	[174,175]
Attention Mechanisms	Focus dynamically on the most relevant input regions, enhancing spatial dependency modeling for tasks like classification, segmentation, and detection.	[176,177,178,179,180]
Self-Attention	Captures long-range dependencies by relating all elements within a sequence, boosting spatial and temporal understanding for image processing tasks.	[181]
ViTs	Treat images as sequences of patches and use self-attention mechanisms for global dependency modeling and scalability with large datasets.	[182,183,184]
Hybrid Architectures	Combine CNNs for local feature extraction with transformers for global context modeling, achieving improved performance for complex image tasks.	[185]
GANs	Involves a generator and a discriminator in an adversarial process to synthesize realistic synthetic images, with applications in style transfer and super-resolution.	[186,187,188,189,190]
WGANs	Introduces a stable loss function to mitigate mode collapse and training instability issues in GANs.	[191]
CGANs	Enable controlled image generation using auxiliary information, improving specific task performance like cross-domain synthesis.	[192]
GANs challenges	Limitations such as training instability, mode collapse, and computational resource demands, emphasizing the need for careful design.	[193,194,195]
Hybrid Models	Combine CNNs with transformers to leverage local feature extraction and global dependency modeling for tasks like video analysis and visual question answering.	[196,197,198,199,200,201]
Multi-Modal Models	Integrate visual, textual, auditory, or sensory data to enhance understanding and decision-making in tasks like medical diagnostics and autonomous driving.	[202,205,206]
Cross-Modal Transformers	Employ transformers for fusing multiple modalities, such as infrared and visible image fusion, enhancing model adaptability and performance across data modalities.	[203]
Co-Attention Fusion Networks	Focus on aligning multi-modal data streams for specific tasks like multimodal skin cancer diagnosis, improving feature integration and decision accuracy.	[204]

**Table 12:** Grouped evaluation metrics.

Category	Metric
Classification	Accuracy Precision Recall (Sensitivity) F1-Score AUC-ROC Log Loss
Segmentation and Detection	IoU Dice Coefficient Jaccard Index Pixel Accuracy
Image Quality	SSIM PSNR NCC
Object Detection Metrics	mAP
Agreement	Cohen’s Kappa MCC
Advanced Evaluation	Balanced Accuracy FID

**Table 13:** Classification of references related to application fields of DL in image processing.

Topic	References	Description
Medical Imaging	[213,214,215,216,217,218,219,220,221]	Discusses the revolutionary impact of CNNs on medical diagnostics, such as cancer detection, Alzheimer’s, and diabetic retinopathy. It also highlights how DL models aid in treatment planning and patient monitoring.
Health monitoring	[222,223,224,225,226,227,228]	Continuous health monitoring using wearable devices and self-supervised learning and AI, addressing challenges such as bias and disparities in diagnostic accuracy across different demographic groups.
Autonomous systems	[229,230,231,232,233,234,235]	Discusses DL applications such as object detection, lane-keeping, and obstacle avoidance in self-driving cars, focusing on real-time decision-making systems like ADASs.
AI and edge computing in autonomous systems	[236,237,238,239,240]	Recent advancements in real-time AI and edge computing enhance autonomous systems’ efficiency.
Remote Sensing and Environmental Monitoring	[241,242,243,244,245,246,247]	Discusses the applications of DL in analyzing satellite and aerial imagery, particularly for tracking deforestation, wildlife, damage assessment from natural disasters, and predicting crop yields.
Enhancing Environmental Monitoring	[248,249,250,251,252,253]	Highlights the integration of DL with remote sensing for environmental decision-making, as well as challenges like the computational cost of processing high-resolution images.
Security and Surveillance	[254,255,256,257,258,259,260]	Pertains to the role of DL in real-time video surveillance, facial recognition, and anomaly detection.
Security Surveillance and Ethical Concerns	[261,262,263,264,265,266,267]	Discusses the ethical implications of deploying DL in surveillance systems, including concerns around privacy, the potential misuse of technology, and bias.
Art and Cultural Heritage	[268,269,270,271,272,273]	Describe the applications of DL in restoring damaged artwork, colorizing old photographs, and digitizing cultural artifacts.
AI Collaboration in Cultural Preservation	[274,275,276,277,278]	Focuses on interdisciplinary collaboration between art historians and AI researchers to ensure DL respects cultural integrity and enhances public engagement.
Ethical and Social Considerations	[279,280,281,282,283,284,285,286,287,288,289,290,291,292]	Addresses bias in DL models, particularly in medical imaging and surveillance, as well as the need for fairness, transparency, and accountability in AI systems and privacy-preserving algorithms.
Interdisciplinary Collaboration	[293,294,295,296,297,298,299,300,301,302,303,304]	Highlights the importance of collaboration between AI researchers and domain experts in healthcare, environmental science, and security for advancing DL applications.

**Table 14:** Classification of references related to DL-based challenges in image processing.

Challenges	References
Data scarcity, particularly in medical imaging, autonomous vehicles, and satellite imagery	[305,306,307,308,309,310]
Computational complexity of DL models and the challenge of deployment on edge devices	[311,312,313,314,315,316]
Interpretability challenges, especially in healthcare, finance, and law	[317,318,319,320,321,322]
Generalization and robustness challenges in DL models	[323,324,325,326,327,328]
Ethical implications, including bias and privacy concerns in AI systems	[329,330,331,332,333,334]

**Table 15:** Classification of references related to DL-based future directions in image processing.

Future Directions	References
Self-supervised learning and data scarcity	[335,336,337,338,339,340]
Efficient model architectures, NAS, pruning, quantization, distillation, neuromorphic and quantum computing	[341,342,343,344,345,346]
XAI and methods for making DL models interpretable	[347,348,349,350,351,352,353]
Integration of emerging technologies with DL, quantum computing, and edge computing	[354,355,356,357,358,359]
Development of new evaluation metrics, fairness, and ethical considerations	[360,361,362,363,364,365]
Opportunities for innovation in self-supervised learning, efficient model architectures, explainable AI, and emerging technologies	[366,367,368,369,370,371,372]

## List of Abbreviations

The following abbreviations are used in this manuscript:

**Acronym**	**Meaning**
AI	Artificial Intelligence
DL	Deep Learning
ML	Machine Learning
GPUs	Graphics Processing Units
CNN	Convolutional Neural Network
ResNet	Residual Network
DesNet	Densely Connected Convolutional Network
FCN	Fully Convolutional Network
R-CNN	Region-based Convolutional Neural Network
YOLO	You Only Look Once
NN	Neural Network
ConvNext	Next Generation of Convolutional Networks
ViT	Vision Transformer
GAN	Generative Adversarial Network
CGAN	Conditional GAN
WGAN	Wasserstein GAN
FGSM	Fast Gradient Sign Method
PGD	Projected Gradient Descent
MAT	Model-based Adversarial Training
UPGD	Universal Projected Gradient Descent
HTPL	Hierarchical Transfer Progressive Learning
RL	Reinforcement Learning
SimCLR	Simple Framework for Contrastive Learning of Visual
DDC	Deep Domain Confusion
DICA	Domain-Invariant Component Analysis
DANN	Domain-Adversarial NN
MANN	Memory-Augmented NN
SNAIL	Neural Attentive Meta-Learner
MAML	Model-Agnostic Meta-Learning
LSTM	Long Short-Term Memory
SGD	Stochastic Gradient Descent
CLIP	Contrastive Language-Image Pretraining
PRISM	Promptable and Robust Interactive Segmentation Model
MoCo	Momentum Contrast
NAS	Neural Architecture Search
TP	True Positive
TN	True Negative
FP	False Positive
FN	False Negative
TPR	True Positive Rate
FPR	False Positive Rate
IoU	Intersection over Union
AP	Average Precision
mAP	Mean AP
SSIM	Structural Similarity Index
FID	Fréchet Inception Distance
PSNR	Peak Signal-to-Noise Ratio
NCC	Normalized Cross-Correlation
ROC	Receiver Operating Characteristic
AUC	Area Under the ROC Curve
MCC	Matthews Correlation Coefficient
ADAS	Advanced Driver-Assistance System
XAI	Explainable AI
